# Measuring scientific impact beyond academia: An assessment of existing impact metrics and proposed improvements

**DOI:** 10.1371/journal.pone.0173152

**Published:** 2017-03-09

**Authors:** James Ravenscroft, Maria Liakata, Amanda Clare, Daniel Duma

**Affiliations:** 1 Centre for Scientific Computing, University of Warwick, Coventry, United Kingdom; 2 Department of Computer Science, Aberystwyth University, Aberystwyth, United Kingdom; 3 School of Informatics, University of Edinburgh, Edinburgh, United Kingdom; GERMANY

## Abstract

How does scientific research affect the world around us? Being able to answer this question is of great importance in order to appropriately channel efforts and resources in science. The impact by scientists in academia is currently measured by citation based metrics such as h-index, i-index and citation counts. These academic metrics aim to represent the dissemination of knowledge among scientists rather than the impact of the research on the wider world. In this work we are interested in measuring scientific impact beyond academia, on the economy, society, health and legislation (comprehensive impact). Indeed scientists are asked to demonstrate evidence of such comprehensive impact by authoring case studies in the context of the Research Excellence Framework (REF). We first investigate the extent to which existing citation based metrics can be indicative of comprehensive impact. We have collected all recent REF impact case studies from 2014 and we have linked these to papers in citation networks that we constructed and derived from CiteSeerX, arXiv and PubMed Central using a number of text processing and information retrieval techniques. We have demonstrated that existing citation-based metrics for impact measurement do not correlate well with REF impact results. We also consider metrics of online attention surrounding scientific works, such as those provided by the Altmetric API. We argue that in order to be able to evaluate wider non-academic impact we need to mine information from a much wider set of resources, including social media posts, press releases, news articles and political debates stemming from academic work. We also provide our data as a free and reusable collection for further analysis, including the PubMed citation network and the correspondence between REF case studies, grant applications and the academic literature.

## Introduction

Research councils and investors now expect research scientists to plan for and demonstrate the impact of their work. Attempting to understand and maximise the impact of research should be beneficial to scientists, not only as a requirement of funding, but also because it would help increase our understanding in terms of how scientific findings can benefit human society and the environment.

### Definitions of impact

Academic impact is considered to be the impact that scientific research has within the academic sphere. Academic impact is traditionally measured through the use of author metrics such as per-author and per-journal citation counts. Slightly more complex impact factors include the Journal Impact Factor (JIF), devised by Garfield in 1955 [1], and the author h-Index devised by Hirsch (2005) [2]. These remain *de facto* success metrics for academic prevalence across the scientific community at the time of writing.

We define the term *comprehensive impact* as the broad impact of scientific research upon human society (including cultural and economic impact) and the natural environment.

Although academic impact is relatively well understood through existing metrics which are further discussed below, comprehensive impact remains very difficult to detect, understand and measure.

### Academic impact metrics

As discussed above, there are already a number of well understood and broadly used metrics for the measurement of academic impact. These metrics have faced a range of criticism from a number of sources.

The simplistic strategy of counting a paper or author’s citations and comparing this sum with the total number of citations that their peers received may seem attractive. However, it is not fair to compare publications from different years or scientific disciplines since there is a great deal of difference in speed and frequency of citation accumulation across different fields of science [3] [4]. Mean Normalised Citation Score (MNCS) is a metric designed to address these shortcomings by normalising for year of publication and scientific sub-field. The “expected” number of citations for a given paper is determined by taking the mean of citations for papers in the same field published in the same year. The MNCS value is the ratio of the actual number of citations a paper received in comparison to the “expected” citation count [4]. An author’s overall contribution could be calculated by taking the average of their MNCS scores for all of their papers. However, this approach is still prone to skew from papers that are exceptionally popular (with respect to their field and year of publication) and thus it is very difficult to differentiate between authors who have many successful publications and authors who have one or two particularly well known works.

Hirsch’s h-Index is an author-level metric that is able to distinguish between frequent strong publishers and publishers with fewer but more exceptionally popular papers. h-index is defined by Hirsch so that “a scientist has index *h* if *h* of his/her *N*_*p*_ papers have at least *h* citations each, and the other (*N*_*p*_ − *h*) papers have no more than *h* citations each.” [2]. This means that an author can only achieve an h-Index of 50 if they have published at least 50 times and 50 or more of their publications have at least 50 citations each.

There are also a number of higher level metrics for academic citation. JIF (Garfield 2006) is a journal-level metric, calculated by taking the average of the interest in all papers published in a journal [1]. There are a number of providers of JIF and similar, journal-level metrics. Most charge for their data but some such as Elsevier provide free access to JIF data(https://www.scopus.com/sources). One of the main problems with using journal-level metrics to understand academic impact is that it is very difficult to derive the source of impact down to a particular institution or even scientist. Furthermore, JIF-style metrics are highly susceptible to skew from successful or unsuccessful outlying papers and since data is usually reported at a high level, it is often impossible for an observer to rationalise or indeed reproduce scores independently [5]. Additionally, research has found JIF and h-Index can be manipulated through self-citation [6, 7].

Citation-based academic impact metrics, such as those discussed above, aim to measure the popularity of journals and authors at a high level, rather than represent the merits of a single piece of research. These metrics are based upon the assumption that influential works or scientists are cited more often than others [6, 8] but Cronin (1984) suggests that citation behaviour may be driven by a number of motivators including the negation or correction of some part of the cited work [9, pp 35–46]. Bornmann (2008) suggests that variation of citation behaviour across disciplines may also mean that comparisons made via h-index and JIF across scientific domains are invalid [10]. A number of scientific bodies and academics have published public warnings on the use of these metrics as benchmarks for academic success [11–13]. Declarations such as DoRA (http://www.ascb.org/dora/) and the Leiden Manifesto [14] comment on the shortcomings of current bibliometrics and provide guidance to institutions on the measurement of their academic staff. Both the Leiden manifesto and DoRA criticise overly complex metrics and try to promote openness and transparency around metadata used to generate scores so that they can be easily independently verified.

### Altmetrics and social based metrics

A number of recent attempts to solve these problems have been suggested. One of the most successful of these has been the idea of “altmetrics”, or alternative metrics, based on evidence from the social web [15, 16]. There exist a number of systems supporting this notion: Altmetric is an organisation which uses a publication’s online footprint (Twitter mentions, Facebook posts and shares etc.) to score its impact [17]. Impact Story (http://www.impactstory.org/) is an online service that provides a combined view of academics’ citations and social media footprint in order to try to provide meaningful context around a person or institution’s academic impact. The service generates profiles for researchers automatically using their ORCID ID [18] and “gamifies” scientific impact by awarding authors with badges that represent milestones in impact. Semantic Scholar (http://www.semanticscholar.org/), whose primary function is as a research search engine, also offers some novel features to enable academic impact monitoring, such as citation importance classification [19] and graphs of citation velocity (how many citations a work receives per month) and acceleration (change in citation velocity). Work by McKeown *et al* [20] has explored the use of sophisticated Natural Language Processing technologies to extract information from the full text of academic papers in order to track the impact of a new term on the community, such as ‘microRNA’. This allows a better measure of academic impact than the cruder citation measures.

Each of the systems discussed above provide a greater understanding of the academic impact of publications than simply using citation count, h-Index or JIF. It could even be argued that Altmetric and ImpactStory begin to provide some insight into the socio-economic impact of publications by examining online social footprints. However, none of them facilitate understanding socio-economic impact in depth. In addition to this, it is possible to game the Altmetric system through the generation of false likes and mentions [21], adding significant noise.

The altmetrics manifesto (http://altmetrics.org/manifesto/) claims that “mature altmetric systems *could* be more robust, leveraging the diversity of altmetrics and statistical power of big data to algorithmically detect and correct for fraudulent activity.” However we believe that these systems are far from mature. Publications on this subject suggest that current systems represent “initial efforts” and Altmetric state that they are ‘still finding [their] way” where understanding altmetric gaming is concerned (https://www.altmetric.com/blog/gaming-altmetrics/)

Bornmann (2016) suggests that the 10 principles of the Leiden Manifesto, originally written to address citation-based metrics, also apply to the use of altmetrics to measure academic impact [22]. Since both citation-based metrics and altmetrics measure the ‘attention’ that publications receive (number of citations, social media ‘likes’, ‘retweets’ etc.) as a proxy for the success of that publication, it is to be expected that they share many of the same strengths and weaknesses.

In order to better understand the different types and extent of comprehensive impact generated by scientific publications and discoveries, it is clear that a different approach is required.

### Comprehensive impact projects

Pointers on how to ascertain scientific impact beyond academia can be found in the STAR METRICS programme in the United States. STAR METRICS aims to build a platform and tools that records where federal funds are invested in research and “offers the scientific community the opportunity to be proactive and to augment anecdotes about the value of science to the nation’s health, security, and economic vitality” [23]. In its current incarnation, STAR METRICS is primarily concerned with understanding scientific impact on employment in terms of how investment in science funds job creation [24]. In the future STAR METRICS will also describe the impact of science on employment, society, and the economy by investigating factors such as health outcomes, student mobility and tracing patents and new company startups [24].

A more UK centric initiative that places great credence upon the importance of scientific impact beyond academia is the Research Excellence Framework (REF). REF is an assessment system for evaluating the quality of research conducted at UK institutions, designed to highlight examples of good scientific research and to demonstrate examples of a variety of different impact types through the publication of qualitative impact case studies. Quantitative metrics such as the ones discussed above are not currently used in the context of REF.

REF defines impact as “effect on, change or benefit to the economy, society, culture, public policy or services, health, the environment or quality of life, beyond academia [25, p. 26].” This definition is very close to our definition for comprehensive impact and as such REF impact scores can be considered a close proxy for comprehensive impact. Each REF submission must include an impact case study for assessment purposes. These case studies are published in plain text and made publicly available via the REF website. In a REF case study, authors typically provide details of the type of impact obtained as well as the names of external sources that can corroborate the impact, for example industrial partners who have directly benefited as a result of the research [25, pp. 27–30].

Despite representing a step in the right direction for understanding a diverse range of impact types, the REF still suffers from a number of shortfalls. The assessment process is resource intensive, requiring a committee of academics to evaluate each submission individually, necessitating brief 3–5 page submissions [25, p. 51]. Thus reports tend to focus on a small number of high impact works from institutions, penalising academics who contribute in small amounts to a large number of projects. Whilst REF does contain provisions for interdisciplinary researchers, typically submissions are only assessed by one Unit-of-Assessment sub-panel (a panel that judges work deemed to be in a similar academic discipline e.g. Physics, Mathematics, Biology and so forth) [25, p. 15] placing great importance on employing diverse assessment panels who can fairly judge interdisciplinary work. The subjective nature of human assessors, combined with flexible guidelines and varying opinions on what makes a good case study is also a potential weakness of this system. Despite the shortcomings of REF impact case studies, they are the closest we have to assessing the impact of scientific work beyond academia and in the following we use these as a gold standard.

### Research objectives

We aim to identify whether existing metrics of academic scientific impact correlate with impact that goes beyond academia, to encompass the society, the economy and the environment.

To this effect we assess whether there is a correlation between the REF impact scores for case studies and existing citation-count-based and social-web-based metrics for the papers and authors referred to in the case studies.

We also investigate whether these metrics could be used to build a predictive machine learning model for REF impact Score.

We also make suggestions and recommendations on future directions in ascertaining comprehensive scientific impact.

## Materials and methods

To enable the analysis of existing impact metrics in relation to the REF impact case studies we perform the following steps:

- We collect all impact case study data from the REF 2014 assessment and extract structured information such as Institution and Unit of Assessment.
- We build up a large collection of scientific publication metadata that lists titles, authors and citation relationships between papers forming a citation network which we call Experimental Citation Network (ECN).
- We look for links between REF impact case studies and papers in the citation network.
- We use our ECN to calculate calculate citation-based metrics for REF related papers and external sources to calculate social-media-based impact metrics.
- To visualise the relation between REF impact scores and impact metrics, we plot calculated impact metrics against known REF impact score. We also calculate correlation scores between the two.
- Finally we construct and evaluate a machine learning regression model using the calculated impact metrics as input and REF impact score as output.

We now describe our choices of data sources, the data model created, the text processing we used to extract the relations for the model from the data sources and our use of the REF impact case study scores.

### Data sources—REF impact case studies

REF make available their entire back catalogue of impact case studies (6637 case studies), via their website (http://impact.ref.ac.uk/CaseStudies/). These were downloaded via an automated process and stored both in a relational database for complex querying and in Apache SOLR for full text search.

A Unit of Assessment (UoA) is a subject domain/classification into which REF impact case studies are grouped for assessment purposes, for example “Computer Science and Information Technology” or “Clinical Medicine.”

The REF process mandates that multi-disciplinary research must still be submitted under a single UoA. Assessment panels are permitted to consult each other in the case of multi-disciplinary work. However, the final impact score is allocated with respect to the UoA that the work was submitted under.

REF impact case study scores are released on a unit-of-assessment-per-institution basis (where a single department/UoA at an institution may submit multiple studies based on the REF guidelines [25, p. 28]). Impact scores allocated to these studies are on a scale from 4* (excellent) down to 1* (poor) or unclassified if the work was not deemed to have any impact. To avoid institutions taking unfair action against academics with low quality REF results, per-case study results are not made available. Instead, for each UoA and institution, the percentage of case studies that have been deemed to be in each of these 5 classes of impact is provided. This makes reporting results on a scale more granular than “per UoA per Institution” (e.g. “Computer Science at University of Warwick”) meaningless since we cannot know the impact score of any individual Case Study. Therefore for each of our experiments in this paper, we calculate scores for the metric under examination for each REF case study. Then we work out the mean average score for said metric per UoA per Institution. For example, the average score for all REF case studies submitted from Computer Science in Warwick would count as one data point and all REF case studies for Computer Science in Aberystwyth as another and so forth. Since case studies can only be submitted to one UoA, these per UoA per Institution result groupings can be considered disjoint.

### Data sources—scientific papers

There are a large number of online sources for academic papers and related metadata that could be processed into citation networks. The popularity of open access publishing means that there are many archives full of scientific papers free to download. Some sources such as Web of Science (http://ipscience.thomsonreuters.com/product/web-of-science) even provide their own citation networks that can be consumed automatically. We aimed to use open access sources where possible to maximise reproducibility. We also aimed to generate citation networks that are as large as possible and relatively diverse rather than rely on individual journals, many of which address very specific areas of research (such as yeast cultures in Biology or deep neural networks within Computer Science). We wanted to collect data that covers entire scientific domains where possible. Large diverse citation networks should allow us to calculate citation metrics accurately for the REF studies under examination. Research aggregators such as CiteSeerX, PubMed Central and arXiv collect open access publications from across broad scientific domains (Computer Science, Biology/Medicine and Mathematics/Physics respectively). This makes them prime sources for building and collecting large citation networks that cover these scientific domains comprehensively.

Snapshots of citation networks from RefSeer [26] (which uses a snapshot of CiteSeerX data) and Paperscape [27] were both downloaded and integrated into the data model. CiteSeerX primarily contains papers relating to computer science and information technology, Paperscape is a citation network built from arXiv, an open access research aggregation service that hosts mostly papers concerned with mathematics and physics. These citation networks contain approximately 5.3M and 903K papers respectively.

A citation network for the PubMed Central open-access collection, mainly concerned with indexing biology and medical papers, was also generated by downloading all papers from the website(https://europepmc.org/downloads/openaccess) and indexing all paper titles and authors in a SOLR index (1.2M papers). Then, reference strings from other papers were used as search queries to find citations within the collection as discussed below.

The RefSeer citation network does include comprehensive citation links between papers and self citations. However, author information for the RefSeer papers was not made available as part of the snapshot, which meant that we were initially unable to calculate per-author h-index for the RefSeer citation network. We used Sickle(http://sickle.readthedocs.io/en/latest/) to scrape the CiteSeer Open Archives Initiative (OAI) repository which contains all paper metadata and match the known paper IDs to their respective authors. This information was stored in the relational database system along with the initial citation network.

We henceforth refer to the citation networks assimilated and augmented from CiteSeer, arXiv and PubMed Central for the purpose of this study as the Experimental Citation Networks (ECNs).

### Data sources—altmetrics

In addition to citation based metrics we also choose social web metrics of impact. We investigated multiple providers of social altmetrics in our introduction. However, we chose to use Altmetric as our source as they provide an easy to use API which takes a paper’s PubMed or DOI identifier and produces a continuous score which could be plotted against REF and used in regression without any further manipulation.

Rather than trying to get the Altmetric scores for every paper in our ECNs, we used the links uncovered between REF studies and papers to identify papers of interest and retrieve Altmetric scores for only this subset of papers. For PubMed and arXiv ECNs this was relatively easy because Altmetric provides a REST API for retrieving scores for papers with PubMed and arXiv publication IDs. For the CiteSeer ECN data, this was more difficult. We had to identify Digital Object Identifiers (DOIs) for each of the RefSeer papers via an online reference aggregator service, CrossRef (http://www.crossref.org/).

We checked that the titles of the RefSeer papers and those suggested by CrossRef matched word for word before accepting the CrossRef suggested DOI.

We were then able to use DOIs directly to retrieve Altmetric scores for these documents.

### Data model

**Fig 1:**
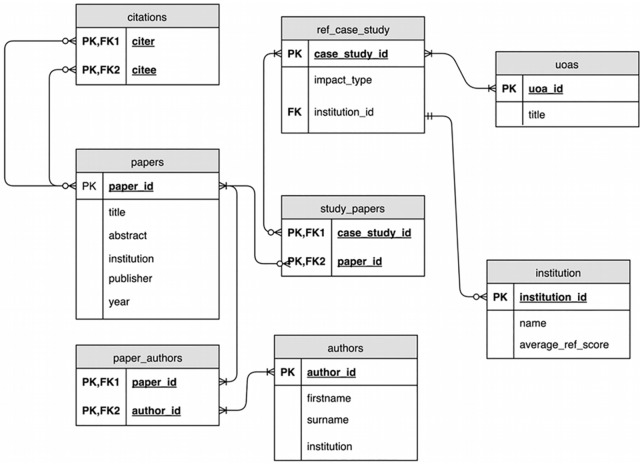
Entity relationship diagram describing how relationships between publications (papers), REF studies, institutions, authors and inter-paper citations.

In the model defined in Fig 1, Papers can have many authors, a title and a year of publication. A self-referential relationship is also defined to allow the expression of citation and the construction of a citation network.

### Linking REF case studies and other data sources

REF case studies contain structured associations with an institution and an area of impact only. No other information linking them to authors or published papers is directly made available as structured data.

REF case studies also provide a section detailing, in unstructured text, related work that contributed to the impact under assessment, which they call “underpinning research”.

**Fig 2:**
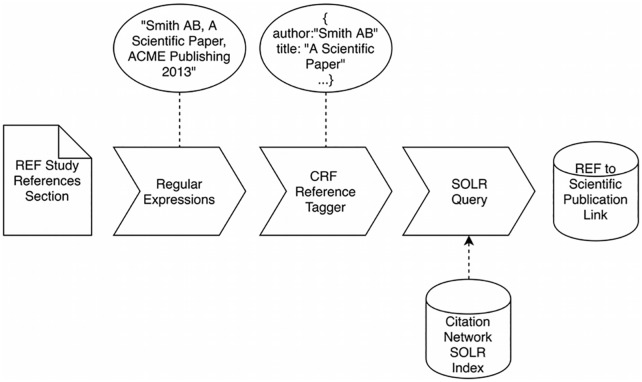
Pipeline process for linking REF studies to publications.

The Underpinning Research section is followed by a Bibliography populated by formal references to the underpinning publications described in the section above. In some cases they also list the RCUK grants that funded the research. These references are usually in the form of a standard bibliography listing that you might expect to find in a scientific publication. In order to exploit this information, a text processing pipeline described in Fig 2 was developed.

The pipeline begins by using regular expressions to pull out strings that are structurally similar to citations from the References section of the case study. Since there are a number of different standards and guidelines for citation syntax depending on field, journal, and even personal preference of the author, a robust way of parsing these citations was required. We used Freecite (http://freecite.library.brown.edu/), a pre-trained Conditional Random Field (CRF) model that does Named Entity Recognition on the title, author, journal name and year of a citation string, making these fields available as a JSON object.

Once parsed with Freecite, the entire reference string from the REF listing is used as a search query in our SOLR indices of the ECNs. Any results are compared field by field with the output from Freecite and if the title and author are a close enough match, a link between the REF case study and the publication is created.

Since we require an exact match between titles normalising for case and punctuation as well as checking that one or more author surnames match between REF citation and paper metadata record, our matching strategy is very conservative and as such we estimate that there are very few errors but also limits our recall for citation matches.

A number of comparison methods were trialled including matching by author and year. However, this caused problems with author ambiguity whereas publication titles are more or less unique across our ECN data set. We also trialled using title sub-string checks and permitted partial matches on titles. Unfortunately this approach was too liberal and falsely matched a number of case studies with papers that had short titles forming part of the reference string.

Table 1 lists the number of links from REF studies to ECNs that could be made.

**Table 1:** Links from REF studies to ECNs.

ECN Source	REF Studies	Papers
arXiv	68	91
CiteSeerX	370	639
PubMed Central	273	322
**Total**	711*	1052

*There are 647 unique studies linked across the three ECNs, some of which appear multiple times giving a total of 711. Paper duplication is explored below.

REF bibliographies are not machine readable and we rely upon pattern matching and machine learning to identify individual references. Therefore, we do not know exactly how many references there are in total and what proportion of them we are able to successfully match. However our regular expression and CRF pipeline returns 6627 references and of these we are able to match 1052 papers from our ECNs.

Note that we deliberately omit Journal Impact Factor(JIF) from our experiment because we do not consider it to be a paper-based or even author-based metric like h-index, citation count and Altmetric score so no direct comparison is possible.

## Results

Our search pipeline was able to identify links between papers in the available ECNs and 647 unique REF case studies. As discussed, it is not possible to gain access to REF impact scores for unique case studies. Therefore, we group results by UoA-per-Institution. This leaves us with 235 result groups (with an average case study population of 2.65 and Standard Deviation of 3.2) to be used in experiments and plotted.

**Fig 3:**
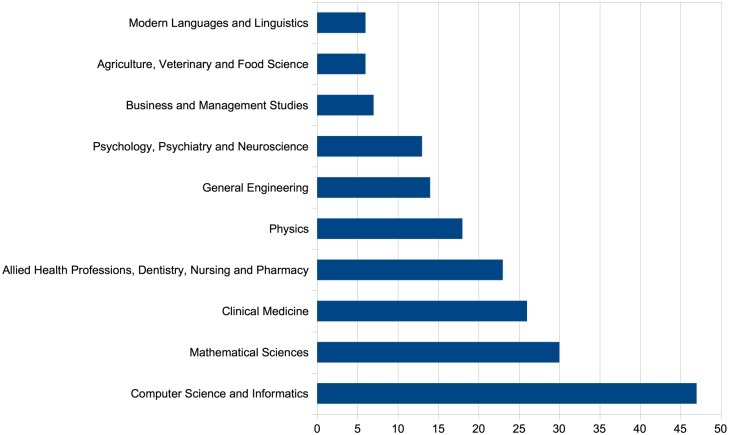
Breakdown of Institution Submissions found to have links to paper in the ECNs per Unit of Assessment. We show here the 10 UoAs with the largest number of ECN-linked submissions only.

Fig 3 below shows the number of institution level submissions per UoA for the top 10 UoAs. The composition of these most frequently identified links is largely as one might expect since the three main ECNs at our disposal are arXiv, which mainly contains Physics, Maths and Computer Science works, CiteSeerX which contains mostly works related to Computer Science and PubMed Central, which mainly contains works in the medical and biological domains.

The predominance of Computer Science papers above all is due to the fact that the CiteSeerX ECN, which only focuses on Computer Science research, is the most homogeneous network among the ECNs we consider. By contrast, papers from PubMed are further distinguished into several categories: Clinical Medicine, Allied Health Professions, Psychology etc. Papers from arXiv can be distinguished into papers from Physics, Mathematical Sciences etc.

### ECN overlap and duplicate papers

Internally the ECNs contain no duplicate papers, however since authors are free to publish their work in more than one journal or repository, there is potential for overlap between ECNS. ECN metadata is quite sparse and there are no computationally efficient means for identifying duplicate papers between networks other than to do a normalised title comparison for every paper title as discussed in Linking REF Case Studies and other Data Sources above.

Since there are of the order of 2 million papers in our ECNs, we restricted the search and comparison to the 1052 papers that have explicit links to REF case studies, as discovered above, and those that cite them. This is sufficient in explaining the extent of ECN duplication within the scope of our study.

We found that of the 1052 papers linked to REF case studies, 63 were duplicate entries. This duplication mainly stems from the PubMed and RefSeer ECNs which both have significant coverage of Bioinformatics publications. We also determined that papers citing the 63 duplicate papers have no overlap. This is the ideal case and shows that our ECNs are relatively complementary in terms of citation network contributions.

In our charts below we plot different impact metrics against Average per-UoA-per-Institution REF impact score for the top 5 of these UoAs, since they have the most data points and are interesting to look at. We provide the full data set for experimentation and further analysis in the appendices and in digital format via Figshare https://figshare.com/s/751679e8993a7fe2c5d8.

### Mean normalised citation score and REF impact score

Our first experiment involved examining the correlation between MNCS and REF case study impact score. MNCS is usually normalised by year and by scientific discipline [4]. The year of publication for papers linked to REF studies was made available to us through the ECN metadata. However, the scientific discipline presented a larger problem since our ECNS each cover broad areas within Biology, Chemistry, Physics and Computer Science. Since we are only dealing with papers that have been explicitly linked to a REF case study in this experiment, we chose to use the REF case study’s UoA as the scientific domain to normalise against. This decision assumes that all papers referenced by a REF case study are from within the same UoA as that REF case study. This allowed us to calculate MNCS using the respective publication years and the UoAs of the papers.

We then average the MNCS scores for each case study to allow us to report by UoA-per-Institution as explained above.

**Fig 4:**
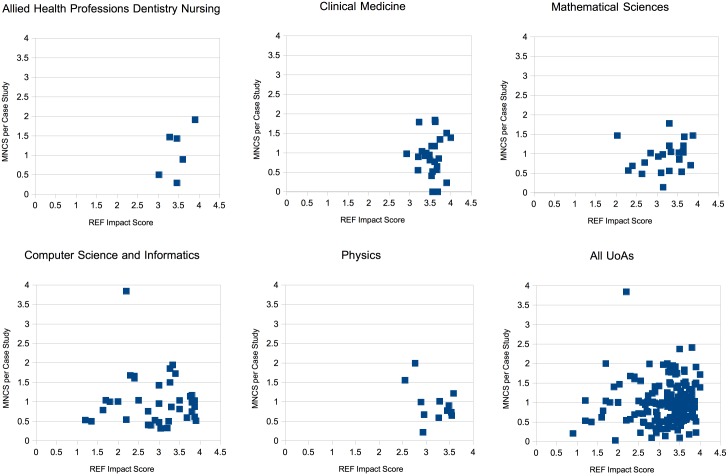
Average MNCS per REF case study vs REF case study score.

MNCS was plotted against average REF impact score and the chart can be seen in Fig 4 below. Visual inspection does not appear to show any clear relationship between the two axes for any of the units of assessment. There is also a very low overall Pearson coefficient of *r* = 0.035 for all UoAs.

### Author h-index and REF impact score

The h-index metric provides a way to measure an author’s total influence in the form of citations but also factor in the number of publications they have made. It acts as a filter on the raw total citation metric, reducing the skew caused by single papers that have a high number of citations. It is simple to calculate: for any given author, their papers are sorted by number of citations in descending order. Then, with *i* papers and assuming *f*(*x*) returns the number of citations for the i-th paper in the sorted list,

h-index is defined by Hirsch as such that “a scientist has index *h* if *h* of his/her *N*_*p*_ papers have at least *h* citations each, and the other (*N*_*p*_ − *h*) papers have no more than *h* citations each.” [2]

In this experiment, we examine the relationship between the average h-index of authors whose work has been cited as underpinning research in a REF case study against the average REF impact score per UoA per Institution.

The h-index values used in this study were generated from the three ECNs. Since these are not as comprehensive as those provided by Google Scholar and Web of Science, we have calculated the h-indices of the most prolific authors in our study and compared these against their respective Google h-index as a probable upper limit.

These are shown in Table 2.

**Table 2:** Comparison of H-indices for Authors: Google vs ECNs.

Author	Google (Since 2011)	Google (All Time)	ECNs
Ellis R. (UCL)	87	144	94
Filippenko A. (University of California, Berkeley)	91	144	72
Jennings N. R. (Imperial College London)	62	107	52
Gächter, S. (Nottingham)	45	49	51
Griffiths T. L. (University of California, Berkeley)	53	62	44
Wooldridge M. (Oxford)	47	82	39
Shawe-Taylor J. (UCL)	40	59	29
Papaloizou, J. (Cambridge)	41	71	20
Merrifield M. (University of Nottingham)	27	43	17
Pourtsidou A. (ICG Portsmouth)	8	8	5

Google Scholar’s author profile page provides two values for h-index, the ‘all time’ value, which is calculated with all papers known to be authored by the person, and ‘since 2011’, which uses the same h-index calculation but on the subset of papers that the author has published since 2011.

The exact numbers of our h-index scores and Google’s h-index scores are rather different. Despite our ECNs being large and relatively comprehensive, it is to be expected that authors featured within them also publish in journals and publications that are not present in these networks but that are present in Google’s indexing platform. Another challenge in this area is author disambiguation which neither our implementation or Google’s implementation of h-index are able to fully address.

For this study, the approximate and relative values for comparison were more important than exact values, which are very difficult to reproduce given the problems described above. We were satisfied that despite the absolute differences between our h-index values and those of Google, the information from the citation network provides enough context to perform relative ranking and measurement within our dataset.

We calculate h-index for all authors of each paper linked to a REF impact study. We then take the mean of h-indices for authors associated with these studies.

All authors are considered with the same weighting and importance across the corpus and no significance is given to the order of author listings on publications in this study.

**Fig 5:**
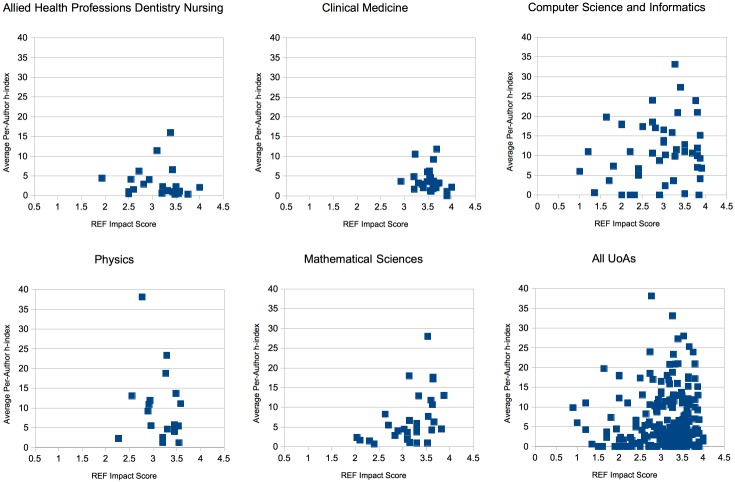
Average per-author h-index vs Average REF Impact Score.

The plot of average author h-index vs REF impact score is shown in Fig 5. This shows very little clear correlation with author h-index scores dotted around almost randomly, which is further validated by a Pearson coefficient of *r* = -0.005 on this dataset.

Our scoped survey does not provide much insight into the extent of duplication across an author’s entire back-catalogue of work. However, in order for duplicate papers to significantly impact an author’s h-index, any paper contributing to *h*, the author’s current h-index, would have to have at least *h* + 1 citations (contributed through novel and duplicate links). We believe that the probability of this happening at a scale significant enough to noticably alter the correlation between Author h-index and REF impact study is very low.

Average author h-index considers authors’ scores independently from the REF case study they co-authored and entirely based upon their entire back-collection of publications and works. This means that it is more accurate to say that plotting author h-index against REF describes the relationship between the historical success of authors and their typical REF scores as opposed to REF scores and their typical academic influence derived from underpinning research. Authors with an excellent historical publication record might end up collaborating on work reported in a low scoring REF case study or vica versa. Especially if authors are interdisciplinary between research areas that are well understood and research areas that are still novel. This may explain the reason for the lack of correlation here.

### Case study h-index and REF impact score

Typically, and in the above section, the h-index metric is calculated per-author. That is, across all publications made by a unique individual. In this study, we treat REF case study as if it were an individual author by calculating h-index over papers mentioned in specific studies that are found in the ECNs. This metric represents the citation-count impact or Academic impact of a REF case study as opposed to the average per-author metric which describes the average academic impact of an author’s entire work. In other words, it more succinctly encapsulates the academic impact of the REF case study than the per-author metric used above.

Our implementation of h-index for a REF case study neatly side-steps the author disambiguation problem since publications are tied to case studies by using exact title matches (with some normalisation for punctuation) between publication and respective REF case studies that list them as underpinning research.

**Fig 6:**
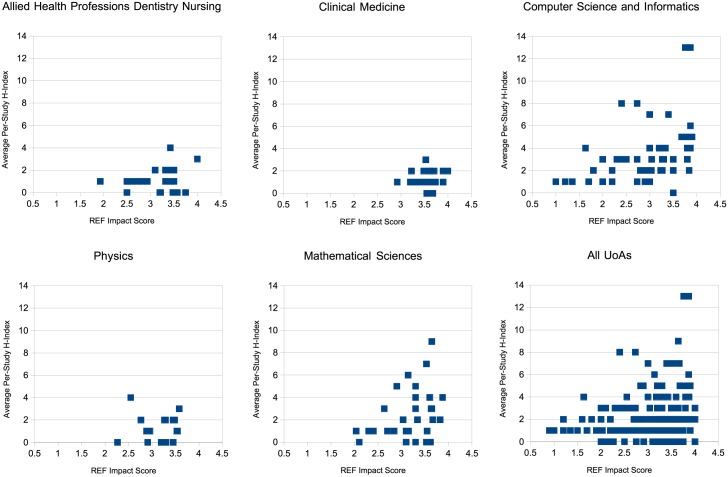
Average per-study h-index vs Average REF Impact Score.

Fig 6 shows a plot of mean average case study h-index versus REF impact score per UoA per Institution for the top 5 UoA categories. Since per-case-study h-index is a metric derived from the pure citation counts depicted in Fig 4, it should come as no surprise that the graph is very similar. However, what is immediately clear from this graph is that there is a more visible correlation between the two metrics (the Pearson coefficient for the whole dataset including the 5 categories shown was *r* = 0.141). This is because h-index acts almost like an averaging filter, reducing the overall effect of a single paper with a large number of citations on any given study.

### Altmetric

Among the existing metrics for academic impact explored above, Altmetric would seem to be the one most closely linked to socio-economic impact since it is modelled upon online social media interactions rather than citations. Altmetric provides a “score” which is a weighted count of online attention that a publication receives. Mentions of scientific works in different sources online such as social media sites (Facebook, Twitter etc) and news publishers (e.g. BBC or The Times) increase an article’s score by a predetermined amount. The full listing of sources and weightings and an explanation of how these are aggregated can be obtained at the Altmetric Website (https://help.altmetric.com/). We used Altmetric API to retrieve scores for as many of the papers linked to REF studies as we could. This search was carried out on 26 August 2016.

**Fig 7:**
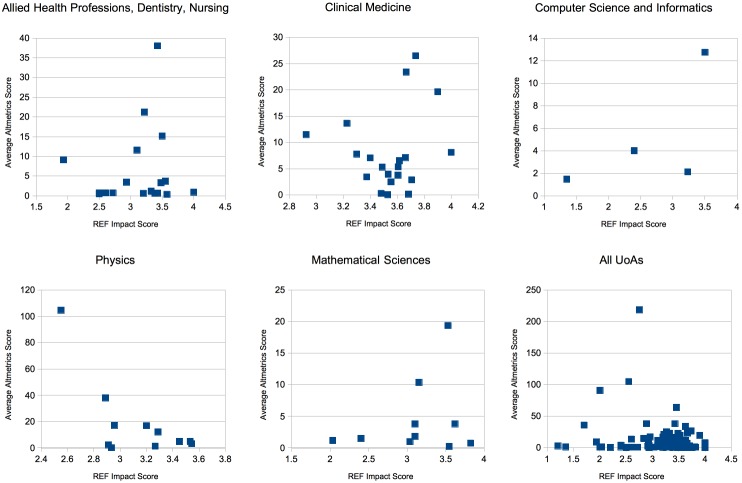
Average Altmetric Score vs Average REF Impact Score. For All UOAs, one outlier is not displayed at (2.75,218).

Unfortunately a large number of the papers under investigation (approximately 40%) had no Altmetric score at all (the API endpoint returned a 404 indicating missing paper profile). Fig 7 shows a graph of the top 5 UoA categories plotted to show Mean Average Altmetric Score per Study versus Impact Score per UoA per Institution.

Perhaps surprisingly the results show that there is little in the way of correlation between Altmetric score and REF impact. The Pearson coefficient for the overall relationship is *r* = −0.0803. This finding seems to support the experience of Thelwall *et al* [28]. Their work investigates the correlation between social data, used by altmetric providers like Altmetric to calculate scores, and citation data. They found that social data coverage was inadequate for drawing any conclusions.

Interestingly, we found that many of the 40% of missing papers appear to have been from Computer Science, which had only 4 samples to display on this graph. This suggests that although Computer Science papers are cited many times by academics (as previous graphs imply), they are not discussed as frequently on social media. A recent study by Costas et al. also found that Altmetrics coverage of Mathematical and Computer Science papers tends to be much lower than disciplines like Biomedicine and Health or Natural Sciences and engineering [29]. Haustein et al. (2014) suggest that papers are more likely to be tweeted if they are “curious or funny, have potential health applications or refer to a catastrophe” [30]. Computer science papers are often abstract or focus purely on a technique or algorithm rather than application. This could explain why computer science papers receive less online attention than biomedical and health papers that have applications that can easily be understood. The inverse appears to be true for Physics and Allied Health Professions papers, which have some of the lower h-index and citation counts in previous graphs but have the highest Altmetric scores. Perhaps these papers address concepts that are more tangible to the public on social media. However, neither of these UoAs demonstrate any kind of correlation between Altmetric scores and REF scores.

### Regression modelling

A summary of the overall Pearson *r* coefficients for each of the metrics discussed above is shown in Table 3.

**Table 3:** Pearson *r* coefficient scores for metrics evaluated against REF Impact score.

UoA	Allied Health Professions, Dentistry, Nursing and Pharmacy	Clinical Medicine	Computer Science and Informatics	Mathematical Sciences	Physics
Papers Linked to UoA	65	72	280	136	52
Mean Normalised Citation Score (*r*)	0.229	-0.172	-0.003	0.182	-0.06
Data Points	16	23	38	23	12
Per-Author h-Index (*r*)	-0.094	-0.21	0.168	0.461	-0.182
Data Points	23	25	42	30	18
Per-Case Study h-index (*r*)	0.178	0.139	0.418	0.347	0.023
Data Points	16	23	46	25	12
Per-Case Study Altmetric Score (*r*)	0.081	0.210	0.058	0.102	-0.426
Data Points	18	20	4	10	11

The *R*^2^ metric used to measure the performance of the regressor can be defined as:

$$R^{2}=1-\frac{SS_{E}}{SS_{T}}$$

where *SS*_*E*_ is the total sum of error squares and *SS*_*T*_ is the total sum of squares.

We implemented a linear regression Ordinary-Least-Squares (OLS) model for predicting REF score of a case study using per-study h-index, per-author-hindex, total citations and mean altmetrics score as features using SciKit-learn [31]. We found that the predictive capability of the model was very poor (*R*^2^ = −0.113). The model was trained using Leave-One-Out cross validation since the data set was too small to sensibly divide up into folds. We also used Leave-Out-One-Feature to understand which of the metrics are most influential in the model. Holding out the Per-Case-Study UoA improves the score the most but none of the scores are useful. Results of this analysis can be seen in Table 4.

**Table 4:** Regression Model Prediction Results.

Features	R2 Score
All Features	-0.113
Without Author H-Index	-0.102
Without Per-Case-Study H-Index	-0.062
Without Per-Case-Study Altmetric Score	-0.067
Without Per-Case-Study UoA	-0.033

We also tried training a Support Vector Regression (SVR) with RBF kernel to see if the aforementioned features could be separated on a hyperplane. However, the *R*^2^ scores for this model were equally uninspiring.

Given the poor and/or inconsistent correlation coefficients for each of the features in this experiment, it is not surprising that a regression model could not be trained.

## Discussion

### Summary

We have demonstrated that there is negligible correlation between REF impact score, as a proxy for comprehensive impact of academic work, and some of the most used academic impact metrics, citation count and author h-index. This supports our theory that REF and the citation-based metrics and altmetrics assessed in this study measure different things. We believe that REF impact is more of a proxy for comprehensive impact whereas citation-based metrics and altmetrics mostly measure academic impact. concerns that earlier researchers have raised around the use of these metrics to in measuring the impact of academic work [11–13]. We describe a way of calculating h-index with respect to REF case studies which offers a stronger correlation than the other metrics investigated in this paper. We do not believe h-index for REF case studies has been calculated before.

However, per-REF-study h-index still leaves much to be desired when trying to approximate the corresponding REF impact score.

Coverage by Altmetric of the papers in our experiment citation networks (ECNs) was insufficient to allow us to draw conclusions regarding the potential of Altmetric scores, but no obvious link between Altmetric score and REF impact score was found. When predicting REF scores from the set of aforementioned metrics as features, neither a simple regression model nor a more complex SVR model was successful.

Despite the huge data collection and sanitation effort, we were only able to identify links between ECNS and a minority of REF studies. We believe this is due to the format of the REF studies which had very varied bibliography styles which were not machine readable. The ECNs were comprehensive in their combined coverage of STEM subjects and the timespans covered too.

However, it is entirely possible that papers referenced by REF case studies were indeed missing from the ECNS. Not all researchers in scientific domains covered by arXiv, CiteSeerX and PubMed Central publish to these repositories. It is possible that our results might have been different had we used Web of Science or Scopus data both of which offer larger, more comprehensive citation networks. On the other hand, it is not clear whether closed access papers would behave any differently to open ones and usage of these closed access citation networks would have prevented us from sharing our data.

Papers need time to accumulate citations. The REF guidelines stipulate that all supporting works must have been published in the ten year window starting in December 2003 and ending in December 2013. The ECN data dumps for Paperscape and Refseer were taken from 2013 and 2014 respectively. The PubMed ECN was generated in August 2016. It is possible that papers published close to the time of generation for each ECN dump have lower citation-based metric scores simply by having had less time to accrue citations and altmetric data. We deliberately use data from close to the REF study period where possible such that we can accurately measure the 10 year window within which the REF report is valid. Notwithstanding, given that we normalised for year of publication in our comparison of MNCS against REF Score, we would expect a stronger correlation of MNCS with REF Score if accrual of citations was a significant limiting factor.

We have made the data used in this paper available on-line to facilitate reproduction of our results and to serve as a starting point for others to experiment with REF impact score prediction. This includes a new citation network (one of the ECNs from the study) for PubMed Central and all processed REF, citation and h-index values discussed in the paper.

Furthermore, we have made recommendations to the REF team of the four UK higher education funding bodies for clearer data representation in any future REF impact case study assessment in order that more accurate analysis, data mining and modelling of comprehensive impact can be made possible. We provide these recommendations in S1 Appendix.

### Conclusions

This study has highlighted the need for further research into methodology development for the detection and measurement of socio-economic impact (comprehensive impact) of academic work and its contributing factors. The REF impact system currently in place is a very manual and resource intensive process. However, we believe that it is a useful “gold standard” for comprehensive impact in use today. Here we have shown that REF impact does offer a perspective on comprehensive impact that citation-based metrics and current generation altmetrics cannot.

We believe that a new, automated methodology inspired by REF impact case studies, taking into account features directly related to comprehensive external impact would be beneficial to both academics and funding bodies. This would support the view taken by McKeown *et al*. that full text features drawn from academic papers are more informative in calculating academic impact [20]. Extrapolating this view, full text features drawn from a number of different textual sources could better represent the underlying information for calculating comprehensive impact. Such an approach would use sources such as traditional media, public debates and policy documents as well as on-line social media posts to help us understand the impact that scientific works have outside the academic sphere.

Altmetric.com and other altmetrics providers already examine some policy documents as part of their offerings. However, there are some concerns about the coverage, or lack thereof, of policy providers with respect to geographical regions and scientific fields [32]. Furthermore, existing analysis of policy documents is based on counting references in these documents to scholarly articles. For papers, we believe that better insights could be drawn by using textual sources to better understand loosely linked works (e.g. via concepts or scientific methods and inventions) and by taking into account the context in which the work is referenced (e.g. is the work referenced because it is being discredited by the WHO in light of new research).

Within this context, REF impact scores could be used as a baseline for a new model for measuring comprehensive impact.

### Vision and future work

We envisage a number of technical challenges in creating a system for recognising and measuring comprehensive impact. Firstly, we will need to gather significant amounts of data of varying types and from a range of sources in order to be able to find reliable patterns that generalise well.

There are likely to be costs associated with data access (e.g. commercial news providers) and storage of large volumes of data. Additional data sources include the RCUK grant database, traditional media content from news outlets and policy documents from the UK Government. We will need to automatically annotate the data collected for various entities and relations and identify links and chains of evidence through time using a variety of Natural Language Processing methods. Some of the key Natural Language Processing technologies that we aim to use and extend in the process of building this system include event detection and open information extraction, stance identification and textual entailment.

This heterogeneous textual evidence collated will provide the features for machine learning methods that can be used to construct models for predicting underlying impact scores.

## Supporting information

S1 Appendix Recommendations for the REF Process. We provide recommendations to REF on how data captured during the next REF Assessment could be optimised for automated analysis.
(PDF)
