# Supplementary material for: Measuring scientific impact beyond academia: An assessment of existing impact metrics and proposed improvements
Source: PLoS One. 2017 Mar 9;12(3):e0173152. doi: 10.1371/journal.pone.0173152 (PMC5344357; doi:10.1371/journal.pone.0173152)
Supplement: S1 Appendix — We provide recommendations to REF on how data captured during the next REF Assessment could be optimised for automated analysis. (PDF) [file pone.0173152.s001.pdf]

S1 Appendix: Recommendations for the REF Process

During our investigation, we found it very difficult to use REF case studies for automatic data mining/analysis purposes. We propose a number of improvements to the REF Case Study format for future assessments so that data mining is easier.

Author Disambiguation

We propose that mentions of specific academics and individuals are always in association with an ORCID identifier. ORCID is a project set up to help tackle author disambiguation by associating every researcher with a unique identifier. The service is free to use, highly scalable and the largest service of its kind currently available. We believe that using ORCID would help researchers more accurately cross-reference cited works, calculate author-based metrics such as h-index and make it easier to fairly attribute impact to those indivividuals who generate it.

Underpinning Research

We propose that the References section of the REF Case Study be more closely standardised. We encountered numerous examples of the References section being used for varying purposes. Most case studies use the References section as a traditional Bibliography, referencing in a standardised way the supporting publications upon which they build their case. However, a number of case studies use the References section as a place to write prose about why each paper they refer to has been included in the study. Even when the References section is being used as a bibliography, the reference style can be inconsistent from one study to the next, even within the same Unit of Assessment.

These factors make it very difficult to easily extract reference information from case studies in order to cross reference them with papers in real citation networks.

We propose that the REF guidelines be updated to make it more clear what the References section should be used for. We also propose that all references be accompanied by a Digital Object Identifier (DOI) where feasible. In an ideal scenario, the REF API could provide structured data containing a list of links to supporting materials.

Contributing Grants

Although unexplored in this study, we also attempted to harvest supporting RCUK grants from case studies in order to build links between grants and resulting impact. We found that the way in which REF studies report supporting grant information is inconsistent and in some cases, studies do not provide this information at all.

Since RCUK make all of their grant proposals public via their Gateway to Research (GtR) interface, we propose that RCUK grants be included into case studies as a structured field containing the unique GtR of each grant that contributed to a study. We propose that there also be the opportunity to declare grants from other funding bodies and independent groups as a separate field.
